# Pollinator-prey conflicts in carnivorous plants: When flower and trap properties mean life or death

**DOI:** 10.1038/srep21065

**Published:** 2016-02-18

**Authors:** Ashraf M. El-Sayed, John A. Byers, David M. Suckling

**Affiliations:** 1 The New Zealand Institute for Plant & Food Research Limited Gerald Street, 7608 Lincoln, New Zealand; 2 Department of Entomology Robert H. Smith Faculty of Agriculture, Food and Environment The Hebrew University of Jerusalem Rehovot, Israel; 3 School of Biological Sciences University of Auckland Tamaki Campus, Building 733 Auckland, New Zealand

## Abstract

Insect-pollinated carnivorous plants are expected to have higher fitness if they resolve pollinator-prey conflicts by sparing insects pollinating their flowers while trapping prey insects. We examined whether separation between flowers and traps of the carnivorous sundew species or pollinator preferences for colours of flowers enable these plants to spare pollinators. In addition, we collected odours from flowers and traps of each carnivorous species in order to identify volatile chemicals that are attractive or repellent to pollinators and prey insects. In *Drosera spatulata* and *D. arcturi*, no volatiles were detected from either their flowers or traps that could serve as kairomone attractants for insects. However, behavioural experiments indicated white colour and spatial separation between flowers and traps aid in reducing pollinator entrapment while capturing prey. In contrast, *D. auriculata* have flowers that are adjacent to their traps. In this species we identified chemical signals emanating from flowers that comprised an eight-component blend, while the plant’s traps emitted a unique four-component blend. The floral odour attracted both pollinator and prey insects, while trap odour only attracted prey. This is the first scientific report to demonstrate that carnivorous plants utilize visual, spatial, and chemical signals to spare flower visitors while trapping prey insects.

Of more than 600 species of carnivorous plants represented in almost every region of the world, about 38% of the species are found in Australia and New Zealand, including several species of sundews in *Drosera*^1^,^2^. Carnivorous plants utilize various trapping mechanisms to capture a wide variety of insect prey that serve as an important nutritional supplement^3^,^4^,^5^. While these plants benefit from insect prey, the plants also need to attract and spare insects that transfer pollen between flowers of individual plants to ensure outcrossing. The dual reliance of carnivorous plants on insects as a source of nutrition and as pollinators can become problematic if feeding on pollinators results in pollen losses and reduces pollen transfer (pollen limitation), which has been defined as a pollinator-prey conflict^6^,^7^. This conflict has been demonstrated in the carnivorous plant *Pinguicula vallisnerifolia* (Grasilla) where frequent capture of pollinators resulted in pollen limitation^8^. It has been proposed that in some species this conflict could be alleviated by significant spatial separation between the trapping surfaces and the flowers or by temporal separation where prey-catching does not function while the plant is flowering^9^,^10^. However, in some carnivorous plants such spatial separation is minimal and temporal separation does not occur, therefore in these cases the two mechanisms would not be effective in minimizing pollinator-prey conflict. In the majority of studies on carnivorous plants with various flower-trap arrangements, there is evidence that the plants tend to spare their pollinators because very few are captured on traps^11^,^12^. This suggests these carnivorous plants are using alternative mechanisms in addition to spatial and temporal separation to accomplish pollinator-prey discrimination^13^,^14^.

Many sundew species of carnivorous plants exhibit an elongated scape with the spatial separation between the distal end of the flower and adjoining the leaf, which functions as a sticky trap. A longer scape increases the mean spatial separation between flowers and sticky traps and has been interpreted as an adaptation to protect pollinators and lessen the pollinator-prey conflict^9^,^10^. On the other hand, Anderson^11^ found no pollinators were captured by two *Drosera* species with either long or short scapes, and flowers on long scapes attracted more insect visitors than flowers on short scapes. Therefore he proposed that sundew have evolved long scapes as an adaption to place their flowers in the flight path of pollinators rather than to protect them from traps. The evolution of long scapes was suggested to be driven by the same selection pressures as those for non-carnivorous plants^15^.

Most plants attract insects to flowers for pollination with visual and olfactory cues^14^,^16^, and there would be strong selection pressure for competing carnivorous and non-carnivorous plants to have evolved similar means for attracting pollinators. Carnivorous plants are dependent on animal-derived nitrogenous nutrients and thus need to attract prey^17^. Ideally, carnivorous plants would attract only prey to their traps and guide pollinators only to their flowers. Any guidance mechanism may entail costs for the plant such as (i) producing unique scents for flowers and traps or (ii) growth to provide greater spatial separation between flowers and traps. Carnivorous plants could evolve specialized chemical mimicry that exploits the chemical communication of insects to attract and capture prey but not pollinators. For example, traps of carnivorous plants might use odours similar to the dead-horse arum flower that mimics odours of a vertebrate host carcass to attract females of blow fly species^18^.

Very little is known about the nature and role of chemical and visual signals that may be involved in inter-specific communication between carnivorous plants and their insect prey and pollinators, or how such signals would resolve pollinator-prey conflicts. Since Darwin^17^ suggested that carnivorous plants attract prey insects, it has become widely accepted that these plants use various cues to attract prey. However, recent studies have only partially addressed these cues and draw conclusions that are inconsistent. For example, visual cues were not deemed important for attracting insect prey in *Drosera rotundifolia* (L.)^19^. In contrast, red colour was found important for attracting prey in *Nepenthes ventricosa* (Blanco)^20^. Several volatile compounds have been identified in headspace of the traps of various species of *Sarracenia*, but no biological activity was ascribed^21^. Therefore, our work is the first to examine both chemical and visual signals as mechanisms employed by carnivorous plants to attract prey and to minimize pollinator-prey conflicts. We hypothesized there may be qualitative and quantitative differences in the volatiles between flowers and traps, perhaps in conjunction with visual and spatial mechanisms, that elicit different responses of pollinators and prey to traps and flowers, and thus optimize fitness for carnivorous plants and their pollinators.

## Results

### Spatial separation of flowers and traps

**Figure 1:**
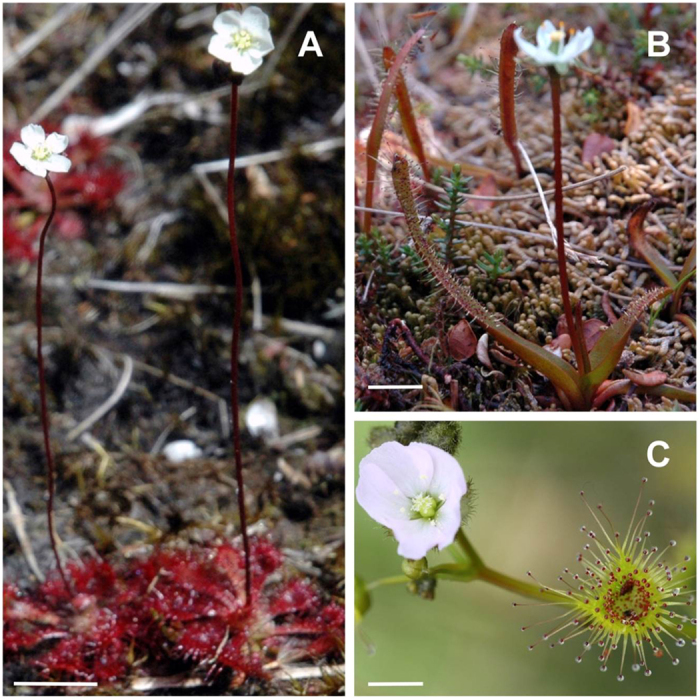
Flower trap arrangement in *Drosera spatulata* (**A**), *Drosera arcturi* (**B**) and *Drosera auriculata* (**C**). Scale bar = 1 cm.

**Figure 2:**
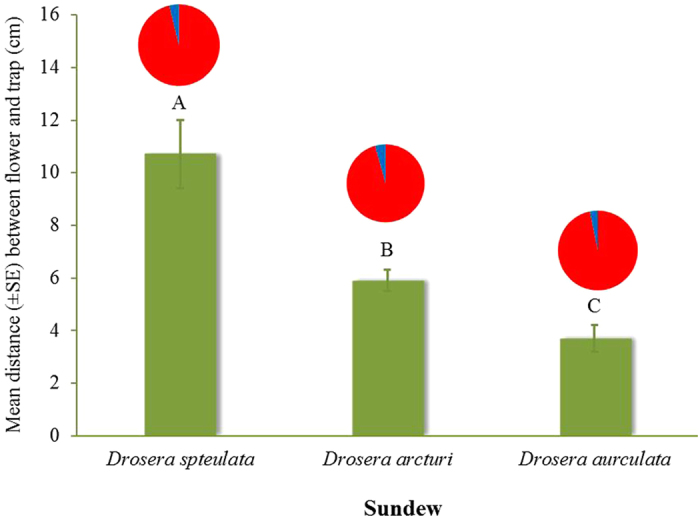
Mean (±SE) distance in cm (*N* = 15) of spatial separation between flowers and traps in the three sundew species. Columns labelled with different letters are significantly different (*P* < 0.001). Pie chart is percentage of flower visitors (blue) of the total number of insects found on traps in the three sundew species.

The three *Drosera* species selected for this study differed with regard to their arrangement of flowers and traps (Fig. 1), with each species having significant differences in the spatial separation between flowers and traps (ANOVA, *F*_2,42_ = 23.8, *P* < 0.001). The flower-trap separation in *D. spatulata* was the greatest followed by *D. arcturi* and *D. auriculata,* respectively (Fig. 2).

### Flower visitors and prey insects

Only Diptera (flies) of the families Syrphidae (hover flies), Tachinidae (tachinid flies), and Muscidae (house flies) were common flower visitors and pollinators of the three carnivorous plants. Syrphidae were the most frequent visitors to flowers of *D. spatulata*, *D. arcturi* and *D. auriculata*, comprising 69%, 72%, and 76%, respectively, of the total number of Diptera (N = 71, 104, and 83) observed on flowers. Tachinidae and Muscidae represented 25% and 6% of the total number of flower visitors for *D. spatulata*, respectively, 14% and 8% for *D. arcturi*, and 14% and 10% for *D. auriculata*. Flower visitors of the three fly families represented 100% (N = 71), 94% (N = 104), and 100% (N = 83) of all insects found on flowers in the three carnivorous species, respectively. Of insects caught by carnivorous traps, there were proportionately few flower visitors of the three fly families representing only 3.8% (N = 183), 4.3% (N = 578) and 3.3% (N = 701) for the three plant species, respectively (Fig. 2). Of the total number of prey (N = 183) found on traps, Hemiptera (bugs), Hymenoptera (wasps/ants), and Coleoptera (beetles) were the most common orders on *D. spatulata*, representing 24%, 19%, and 18%, respectively. Other prey orders included Diptera, Lepidoptera (moths), and Thysanoptera (thrips). Diptera and Coleoptera were the most common prey trapped by *D. arcturi* representing 40% and 23% of the total number (N = 578), respectively. Other prey of *D. arcturi* included Lepidoptera, Hemiptera and Thysanoptera. In *D. auriculata* traps, Diptera were the most common prey representing 48% of the total (N = 701). On *D. auriculata* traps, Sciaridae (dark-winged fungus gnats), Chironomidae (midges), and Culicidae (mosquitoes) were the major dipteran families, representing 61% of total Diptera (N = 335). Hemiptera was the second major group representing 16% of the total number of prey found on *D. auriculata* traps. Less common prey orders included Lepidoptera, Trichoptera (caddisflies), Coleoptera, Hymenoptera and Thysanoptera.

### Escape of flower visitors from traps

Of the 30 dipteran flower visitors placed onto traps of each plant species, 13% (5–30% for 95% B.C.L.), 7% (2–21%), and 20% (9.5–37%) managed to escape the traps of *D. spatulata*, *D. arcturi*, and *D. auriculata*, respectively. No significant differences were observed in the rates of escape among the three plant species (*P* = 0.32, Chi-square).

### Floral and trap volatiles

Analysis of the headspace of the flowers and traps of *D. spatulata*, *D. arcturi* and *D. auriculata* indicated that flowers and traps of *D. spatulata* and *D. arcturi* are scentless, while flowers and traps of *D. auriculata* release odours. We detected eight compounds in the floral headspace collection and four compounds in the trap headspace collection from *D. auriculata* (Table 1). There were no compounds in common between its flowers and traps with each tissue having a unique odour of specific volatiles. 2′-aminoacetophenone (34% of total volatiles) and 2-phenylethanol (30%) were the most abundant scent compounds detected from the flowers, while plumbagin (74.4%) was the dominant compound in the headspace collected from traps of *D. auriculata* (Table 1). Minor constituents (2–14%) in the floral headspace included (+/−)-α-pinene, benzaldehyde, (−)-β-pinene, (+/−)-limonene, benzyl alcohol, and phenylacetaldehyde. In contrast, minor constituents (5–15%) in the headspace of traps included linalool, geranyl acetone and (*E*)-β-farnesene.

### Colour preferences of flower visitors and other insects

**Figure 3:**
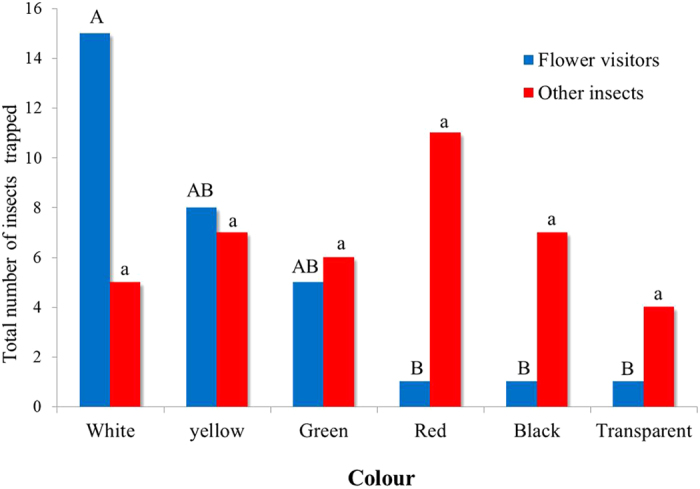
Attraction of flower-visiting insects and other insects to sticky discs of various colours. Bars of either flower visitors or other insects designated with different letters were significantly different (*P* < 0.05).

Visual cues significantly affected preference of the flower visitors to different coloured discs (Fig. 3, two-way ANOVA, *F*_5,20_ = 7.85, *P* < 0.001). A significantly higher number of flower visitors was attracted to white discs than to red, black, and transparent (*P* < 0.05, Fig. 3). There were no significant differences between the numbers of flower visitors attracted to yellow, green, red, black or transparent discs (Fig. 3). Visual cues did not affect colour preference of the non-flower-visiting insects (two-way ANOVA, *F*_5,20_ = 0.68, *P* = 0.64).

### Spatial separation and visual cues in resolving pollinator-prey conflict in *D. spatulata* and *D. arcturi*

**Figure 4:**
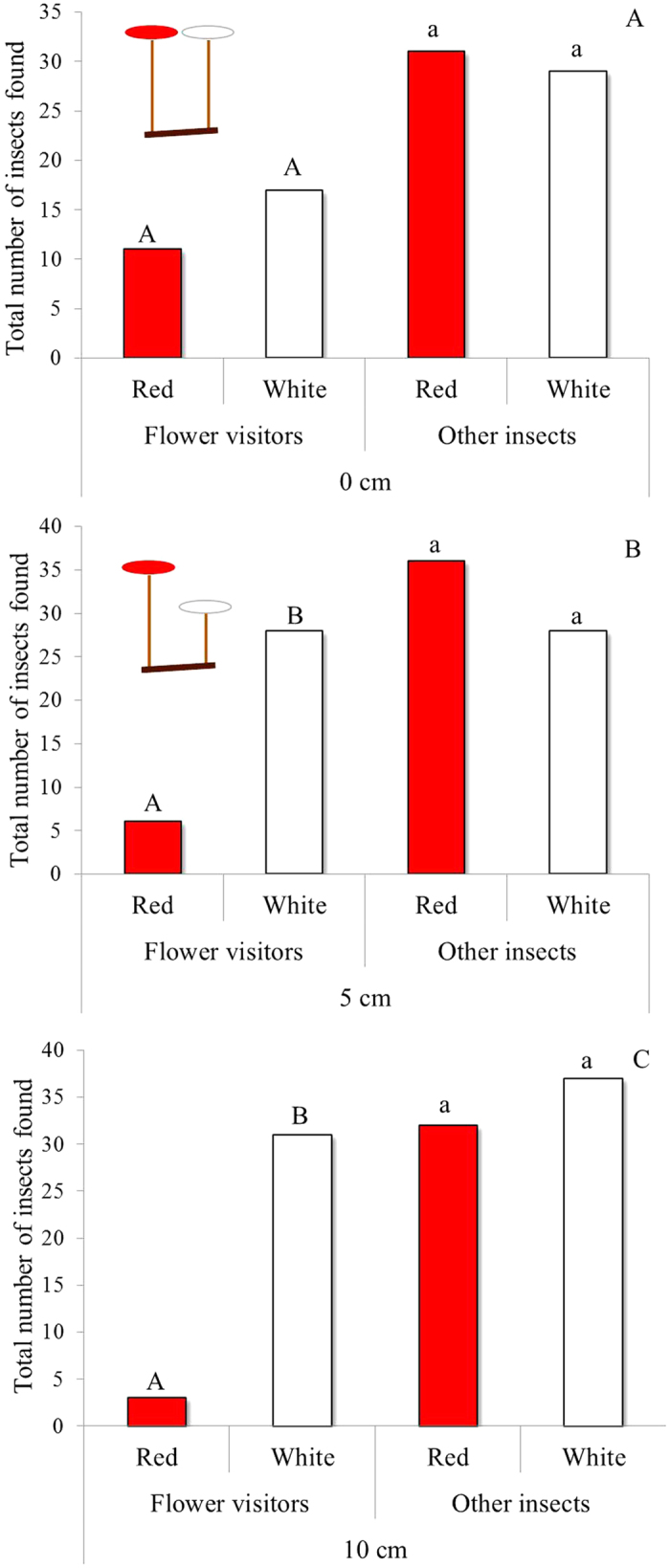
Trapping flower-visiting insects and other insects on red and white sticky discs mounted next to each other in various arrangements. (**A**) Both discs were presented next to each other, (**B**) separated by 5 cm, and (**C**) separated 10 cm. Letters above bars indicate significant differences at 5% level (Exact binomial test).

When the red and white discs were presented side by side, the 28 flower visitors were distributed not significantly different from an equal ratio as tested with the exact binomial test (EBT, *P* = 0.34) with 61% caught on white discs compared with 39% on red discs (Fig. 4A). However, increasing the distance between the red and white discs to 5 cm resulted in 82% of those attracted (N = 34) being caught by the white disc, while further increasing the distance to 10 cm gave 91% landing (N = 34) on the white disc compared to the red disc (*P* < 0.001 and *P* < 0.001, respectively; Fig. 4B,C). On the other hand, non-flower-visiting insects showed no preference for red or white discs when both discs were presented next to each other, or separated vertically by either 5 or 10 cm (Fig. 4A–C).

### Odour in resolving pollinator-prey conflict in *D. auriculata*

**Figure 5:**
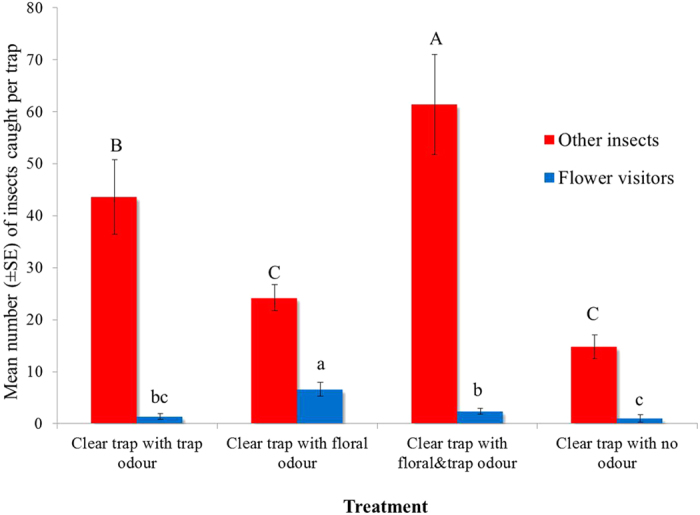
Attraction of flower-visiting insects and other insects to clear sticky cylinder baited with trap odour, floral odour, both floral and trap odour, and no odour. Letters indicate significant differences between means of treatments for other insects or flower visitors at 5% level.

Significantly more flower visitors were attracted to clear sticky surfaces baited with floral odour compared to the same surfaces baited with trap odour or both floral and trap odour (Fig. 5, two-way ANOVA, *F*_3,12_ = 29.4, *P* < 0.001). Thus, trap odour reduces the attraction of flower visitors to floral odour (Fig. 5). A few flower visitors were attracted to clear surfaces baited with trap odour, but this was not significantly different from the control without odour. Significantly larger numbers of other (non-pollinating) insects were captured on clear surfaces baited with floral and trap odour than on traps with either floral or trap odour alone. Trap odour released from clear surfaces captured significantly more other insects than surfaces with floral odour and traps without odour (Fig. 5, two-way ANOVA, *F*_3,12_ = 41.5, *P* < 0.001).

### Colour and odour in resolving pollinator-prey conflict in *D. auriculata*

**Figure 6:**
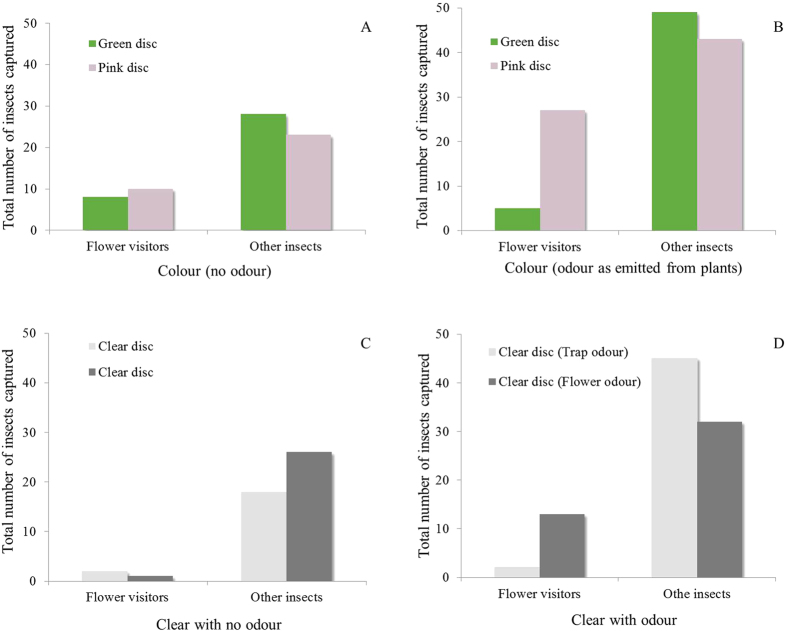
Trapping flower-visiting insects and other insects on green and pink sticky discs mounted next to each other with floral and trap odour in various arrangements.

Flower visitors (N = 18) showed no preference for green or pink colour when the two colours were presented side by side and without odour (Fig. 6A, *P* = 0.81, EBT). In the same experiment, insects other than flower visitors (N = 51) showed no preference for the green or pink colour (*P* = 0.58). The addition of the floral odour to the pink discs and the trap odour to green discs resulted in an increase in the number of flower visitors attracted to the pink discs and a reduction in the number of flower visitors attracted to the adjacent green discs (Fig. 6B, N = 32, *P* < 0.001). In addition, the number of other insects attracted to green discs releasing trap odour was similar to those attracted to pink discs with floral odour (N = 92, *P* = 0.60). Only three flower visitors landed on the clear discs without odour. Forty-four other insects landed on the two clear discs without odour and there was no preference for either disc (Fig. 6C, *P* = 0.29). The number of flower visitors attracted to clear discs with floral odour was significantly higher than the number of flower visitors attracted to clear discs with trap odour (Fig. 6D, N = 15, *P* = 0.01). Similar numbers of other insects were attracted to clear discs with floral odour and to clear discs with trap odour (Fig. 6D, N = 77, *P* = 0.17). The total number of flower visitors attracted to the green/pink discs with odour was significantly higher than the total number of flower visitors attracted to the green/pink discs without odour (Fig. 6A,B, N = 50, *P* = 0.05). More flower visitors were attracted to clear discs with odour than to clear discs without odour (Fig. 6C,D, N = 18, *P* = 0.01). The total number of flower visitors attracted to green/pink discs without odour was significantly higher than the flower visitors attracted to clear discs without odour (Fig. 6A,C, N = 21, *P* = 0.002). The total number of other insects attracted to the green/pink discs with odour was significantly higher than the number of other insects attracted to the green/pink discs without odour (Fig. 6A,B, N = 143, *P* < 0.001). A higher number of other insects were attracted to clear discs with odour than the number of other insects attracted to the clear discs without odour (Fig. 6C,D, N = 121, *P* = 0.003). There was no significant difference in the number of other insects attracted to green/pink discs without odour than to clear discs without odour (Fig. 6A,C, N = 95, *P* = 0.53).

## Discussion

The three sundew species we investigated exhibit different roles for visual and chemical cues as well as spatial separation between flower and traps to lessen the pollinator-prey conflict. In spite of differences in spatial separation between flowers and traps in the three species, only a similarly small proportion of flower visitors were caught in traps of each species. This finding is consistent with previous studies of other carnivorous sundews where prey caught in traps consisted of only a few percent pollinators^11^,^12^. We hypothesized that the small number of pollinators caught in carnivorous traps could be due to either the ability of flower visitors to free themselves (and perhaps learn to avoid traps) or to other mechanisms employed by the plants that spare flower visitors. Our results indicate that a large proportion of flower visitors were not able to free themselves from the sticky secretions. This suggests other mechanisms such as visual, chemical, or spatial/temporal separation between flower and traps may be employed by the plants to minimize trapping of flower visitors. Our results show that flowers of both *D. spatulata* and *D. arcturi* are unusual because no chemicals were detected in their headspaces, suggesting pollinators are attracted to visual cues of flowers while spatial separation between flowers and traps may reduce entrapment of pollinators. In our experiments on colour preference of flower visitors, white caught significantly more flower visitors than red, black or transparent colours. Flower visitors usually show strong responses to visual stimuli^24^. White and yellow are the most dominant floral colours in native New Zealand fauna and syrphid flies show strong preferences for these two colours^25^. It could be argued that since we haven’t examined the pollen load of flower visitors that syrphids and non-syrphid diptrans are not important pollinators. However syrphids and non-syrphid dipterans are well established pollinators especially at high elevations where these plants grow^26^. Both *D. spatulata* and *D. arcturi* have a white flower and these are associated with the non-carnivorous but cohabiting cushion plant, *Donatia novae-zelandiae* (Hooker). The cushion plant is common and produces large numbers of similar appearing white flowers at the same time (Fig. 4S). Interestingly, many floral compounds have been detected in the headspace of the cushion plant (unpublished). This suggests that both sundew species utilize white flowers to visually mimic the flowers of cushion plants and benefit from a common search image of insect pollinators while saving physiological costs of producing scents that could also cost ecologically if herbivores are attracted. Colour and shape mimicry have been reported in the pollination of an orchid^27^.

Because we found that sticky traps of both *D. spatulata* and *D. arcturi* do not release volatiles that could attract prey insects, prey capture by traps is either mediated by visual cues, or by random landing resulting in passive capture. Schaefer and Ruxton^20^ concluded that red colouration is an adaptive trait that increases capture rates for carnivorous plants. However, Jürgens *et al.*^28^ suggested that both pollinators and prey land less on red disc models than on green discs in the sundew habitat of New Zealand. In our field experiments, other insects not visiting flowers showed no preference for various visual cues, suggesting these two plants employ passive sticky traps to catch prey. Similarly, the red colouration of *Drosera rotundifolia* (L.) was not important in the attraction of prey insects^19^. Both *D. spatulata* and *D. arcturi* sundews grow with cushion plants in ombrotrophic open bogs whose soil is nutrient poor, especially in nitrogen. Although sundew carnivorous plants would seem to benefit by using an odour to attract prey to their traps and another odour to guide pollinators to their flowers, producing two different scents could be costly. In open bogs, there are few other plant species (except the low growing, mat-forming cushion plant) that offer landing perches. Thus, passive trapping may be sufficient to capture insects in such environments. Passive trapping is also suggested by the lack of selectivity in prey capture, as shown for *D. spatulata* traps at ground level that mainly captured walking insects of Hymenoptera and Coleoptera, while traps of *D. arcturi* that are just above ground level caught a mixture of flying and walking insects (Fig. 1).

In our experiments, colour alone was not enough to reduce the number of flower visitors caught on red discs representing carnivorous traps when the white and red discs were adjacent. Only when the two coloured discs were separated by 5 or 10 cm did the number of flower visitors land more on the white discs rather than on the red ones, suggesting that spatial separation between flower and trap, in combination with flower colour, is employed by these two species to protect pollinators. Anderson^11^ found no pollinators were caught in the traps of two *Drosera* species with short and long scapes, while the flowers of the species with short scapes received less pollinator visits compared to flowers of the other species with long scapes. He suggested that *Drosera* species evolved long scapes to receive more floral visits rather than to protect the pollinator from traps. Our findings of little overlap in flower visitors and prey on flowers and traps agree with those of Anderson. However, Anderson^11^ did not provide a mechanism by which plants could prevent pollinators from being caught in traps, either by visual or chemical signals, especially in species with short scapes. When the spatial separation between flower and traps was small as in *D. auriculata*, the number of flower visitors caught on traps was still relatively low, suggesting a non-spatial mechanism such as a semiochemical one for protecting pollinators.

Our chemical analysis is the first to identify volatiles released from both flowers and traps of a carnivorous plant, yielding unique odours with no overlap in chemical composition. Floral odour contained 2′-aminoacetophenone and 2-phenylethanol as the main compounds, while trap odour had plumbagin as the main compound. 2-phenylethanol is a well-known floral volatile^22^, while 2′-aminoacetophenone has not been reported as a floral compound but as a volatile from honey^29^. Our synthetic blend of floral compounds attracted both flower visitors and prey insects. In contrast, the synthetic blend of trap volatiles was not attractive to flower visitors but rather attracted prey insects. Floral and trap odours released together resulted in a significant reduction in the number of flower visitors but also a significant increase in the number of prey insects captured. The behavioural activity of specific compounds in the floral and trap blends remains to be determined. Plumbagin has been identified in the extract of the traps of other *Drosera* species^30^ but we show for the first time that the compound is present in biologically-active headspace.

In *D. auriculata*, visual cues alone were not enough to protect flower visitors since both green and pink discs attracted almost equal numbers of flower visitors. In contrast, flower visitors showed a preference for pink discs with floral odour compared to green discs with trap odour. This suggests that odour is the main mechanism employed by carnivorous *D. auriculata* to guide flower visitors to their flowers. Associative learning of odour is well documented in insects^31^. Although visual cues were less important in reducing pollinator-prey conflict, more flower visitors were caught in a trapping model with visual cues than without. Similarly, more flower visitors were caught in a trapping model with both visual and chemical cues compared to a similar model without chemical cues. This suggests an additive interaction between colour and odour in attracting flower visitors to the plants, while odour was critical for the flower visitors to discriminate between flowers and traps. Synergistic interactions among colour, shape and odour in attraction of insects are known. For example, bark beetles exhibit increased attraction to aggregation pheromone when released from various coloured silhouettes resembling trees^32^,^33^. Honeybees are known to more strongly associate rewards with colours when combined with flower fragrances^34^,^35^. Tsetse flies are attracted in higher numbers to dark cylinders the size of vertebrate hosts and emanating CO_2_ and acetone than to the same cylinders without the odours^36^.

The pollinator-prey conflict in carnivorous plants has been defined by Wickler^6^ and Wiens^7^ and demonstrated by Zamora^8^. Our objective was not to demonstrate the pollinator-prey conflict but rather to investigate whether the three *Drosera* species have evolved specific mechanisms to protect pollinators. Previously, two mechanisms have been proposed to alleviate this conflict: a) spatial separation between the trapping surfaces and the flowers; and b) temporal separation where prey-catching is not functioning while the plant is flowering^9^,^10^. In both *D. spatulata* and *D. arcturi,* spatial separation is the main mechanism that reduces the number of flower visitors caught on the trapping surfaces, while visual cues help to guide flower visitors to flowers. In these two species, prey attraction is not mediated by visual or chemical cues; rather passive trapping was the main mechanism to capture prey insects. The lack of ability to attract prey in the two species violates the condition of the carnivorous syndrome in which prey are lured to the traps^10^,^17^,^37^. The relatively few landing perches in the bogs facilitates prey capture by traps, and would provide little selection pressure for traps to evolve attractive chemical cues.

In *D. auriculata* where spatial separation is reduced and temporal separation does not occur, there could be strong selection pressure to evolve another mechanism to minimize pollinator-prey conflict. In contrast to scentless *D. spatulata* and *D. arcturi,* unique odours were identified from the flower and trap headspaces of *D. auriculata* that mediate the attraction of flower visitors to flowers while avoiding sticky traps, whereas trap odours attract prey insects. This species can be considered truly carnivorous because it fulfills the condition of the carnivorous syndrome. Our results report the first example of odour used by carnivorous sundew plants to attract prey insects since first hypothesized by Darwin^17^. Our findings suggest co-evolution between insect pollinators and carnivorous plants in which the latter provide either spatially separated visual cues or distinct odours as an “honest” signal to allow flower visitors to avoid being trapped.

## Methods

### Plants, study sites

The three carnivorous *Drosera* species we studied utilize the same sticky trap mechanism (Fig. 1). *Drosera spatulata* (Labill.) is a small rosette sundew 1.6 to 4.2 cm in diameter with sticky leaves laying flat on the ground. A long 6–20 cm upright scape bears one to five white flowers that open sequentially. *D. arcturi* (Hook.) has linear upright sticky leaves 4–12 cm long and a solitary white flower on a long scape (6–12 cm) positioned immediately above the leaves. *D. spatulata* and *D. arcturi* occur together in alpine and subalpine grassland habitat. *D. auriculata* (Backh. ex Planch.) has a bushy architecture that grows to 100 cm height with up to 12 flowers at different levels that can open next to adhesive traps. Studies of *D. spatulata* and *D. arcturi* were carried out in Arthur’s Pass Canterbury, South Island New Zealand (42°55′0.36″S 171°33′20.09″E). Studies of *D. auriculata* were conducted near Kai Iwi Lakes, (35°48′46.34″S, 173°38′58.36″E) Northland, on New Zealand’s North Island.

### Spatial separation of flowers and traps

The three *Drosera* species showed morphological difference in the flower trap arrangement. To quantify this difference, the distance between the flower and the nearest trap was measured using a ruler (N = 15 for each *Drosera* species).

### Flower visitors and prey insects

For *D. spatulata* and *D. arcturi*, we randomly selected 120 flowering plants of each species and 60 plants of *D. auriculata* for observation of flower visitors. Observations were conducted over several days between 10 AM to 4 PM during the summer flowering periods of each species (October–January, 2009–2012) when insects were abundant on warm, still and sunny days. Insects visiting flowers were captured by net or by hand and transferred to a vial with 75% ethanol and later identified to family or order. Arthropod prey caught in sticky traps of the flowering plants of each species were removed by forceps and transferred to a vial with 75% ethanol and identified similarly.

### Escape of flower visitors from traps

The low proportion of flower visitors in traps found in previous studies could be due to escape and possible learning to avoid traps after escape. To investigate this possibility, a total of 30 undamaged flower visitors captured by net from the dipteran families Syrphidae, Tachinidae, and Muscidae (approximate ratios of 20:5:5 respectively) were placed individually onto a trap of 30 different plants of each of the three carnivorous plant species in the field. If an insect remained on the trap for less than 30 min, it was recorded to have escaped. These tests were done during the same observation times described above.

### Floral and trap volatiles

Volatile collections from flowers or traps of the three species were made both in the field study sites and in the laboratory under natural light and ambient temperature (18–25 °C) by using a dynamic headspace collection method followed by Tenax adsorbent extraction. Ten volatile samples from flowers, ten from traps, and ten control samples were collected from each of 10 individual plants of each species. For *D. spatulata* and *D. arcturi*, volatile collections were conducted in the field from December-January over two flowering seasons (2009/2010 and 2010/2011). For *D. auriculata*, volatile collections of ten samples were conducted in the field from October—November in two flowering seasons (2010 and 2011). All volatile collections were conducted on a sunny days from 10 AM to 4 PM. Additional samples were collected in the laboratory using potted mature plants collected as seedlings from the field. In the dynamic headspace collection (a), the intact flower head or trap was housed in a glass container (i.d. 4.0 cm, 6.0 cm high). The glass container consisted of two parts that tightly closed together using ground glass fittings. One part had a narrow slot (2 mm wide × 17 mm long) to allow insertion of the flower and trap into the glass container without damaging the stem. A charcoal–filtered air stream was pulled over the flower and the headspace was collected on an adsorbent containing 50 mg of Tenax-GR 35/60 (Alltech Associates Inc.) in a 15 mm long × 10 mm diameter glass tube. Tenax adsorbents were thermally conditioned at 200 °C under a stream of nitrogen before use. The airflow in the headspace collection system was 0.5 L/min and each collection session lasted for 6 h. The charcoal filter used to clean the incoming air was thermally activated before use in an oven at 200 °C. Control samples were collected with the above system but without flowers or traps to distinguish between floral and trap compounds and ambient contaminants. Immediately after volatile collection in the field, Tenax adsorbents were sealed in aluminium foils and transported to the laboratory in dry ice. Tenax adsorbents were extracted with 1 mL of hexane (5 × 200 μl aliquots, n-hexane analaR BDH, Laboratory Supplies, Poole, England). Eluted samples were sealed and stored at −80 °C until they were reduced to 10 μl at ambient temperature under a stream of argon immediately before GC/MS analysis.

The concentrated extracts of headspace from flowers and traps were analysed using a Saturn 2200 GC/MS (Varian Inc., Walnut Creek, CA, USA) equipped with a 30 m × 0.25 mm i.d. × 0.25 μm, VF5-MS capillary column (Varian Inc.). The spectra were recorded at an ionization voltage of 70 eV over a mass range m/z of 20 to 499. The transfer line and the ion trap were held at 250 °C and 180 °C respectively. The oven was programmed from 40 °C (held for 2 min), to 240 °C at 4 °C/min. Samples were injected in splitless mode with helium as carrier gas and the temperature of the injector was maintained at 220 °C. Compounds were identified by comparing their mass spectra with authentic standards (Sigma-Aldrich, MO, USA) and NIST 2005 MS library, as well as coincidence with Kovats retention indices published in the literature^22^.

### Colour preferences of flower visitors and other insects

An experiment at Arthur Pass (December 2010—January 2011) was conducted to determine the role of colour in resolving pollinator-prey conflict in *D. spatulata* and *D. arcturi*. Six colours (white, yellow, green, red, transparent, and black) were tested for colour preferences of the flower visitors and prey. White (flower colour of *Drosera* species), yellow, and red are common flower colours, green and red are similar to trap colours, while transparent and black may be less visible to insects. Coloured discs of 20-mm diameter and 3.3-mm thickness were made of smooth-walled polypropylene internally corrugated sheet (Etec, Auckland, New Zealand) coated with sticky polymers (Tanglefoot Company, Grand rapids, MI, USA). Each disc was positioned 10-cm above and parallel to the ground using a wooden stick (3-mm diameter). Transparent plastic discs were included to provide a negative control. Five replicates of each colour were tested by placing them in five rows separated by 3 m such that each row contained one replicate of each treatment (separated 2 m apart) in a randomized block design. Insects were trapped on the discs over 10 d. In order to construct plastic models of carnivorous traps and flowers with appropriate colours, reflectance spectra of the various natural and model colours were measured (Figs 1S, 2S and 3S) using an S2000 spectrophotometer (Ocean optics, FL, USA).

### Spatial separation of visual cues in resolving pollinator-prey conflict in *D. spatulata* and *D. arcturi*

White and red discs (20-mm diameter and 3.3-mm thickness) made of the polypropylene sheet coated with sticky polymers were positioned above the ground using wooden sticks (3-mm diameter) to mimic the visual cues and spatial separations between flower and trap in *D. spatulata* and *D. arcturi*. Pairs of discs were fixed horizontal to the ground with the white disc above the red disc, because white flowers are above red traps in both species, and vertically separated by one of three different distances, 0, 5 or 10 cm. Each of the three separation distances were replicated ten times assigned in ten rows using a randomized block design. Disc pairs of each distance were separated by 1 m in each row, while rows were separated by 3 m (Arthur Pass, December 2011/January 2012).

### Odour in resolving pollinator-prey conflict in *D. auriculata*

Two synthetic chemical blends were prepared that mimicked the flower and trap odours of *D. auriculata*. The floral odour blend contained (+/−)-α-pinene, benzaldehyde, (−)-β-pinene, (+/−)-limonene, benzyl alcohol, phenylacetaldehyde, 2-phenylethanol, and 2′-aminoacetophenone in a ratio of 0.3:0.5:0.7:0.5:1:0.5:3:3.5 (w/w, as in flower volatile collections). The trap odour blend contained linalool, geranyl acetone, (E)-beta-farnesene and plumbagin in a ratio of 1.5:3:1:4.5 (w/w, as in trap volatile collections). Ten mg in total of either synthetic blend was applied in 20 μl of n-hexane GR (Merck Ltd, New Zealand) to a square of black felt (4 mm × 4 mm). Black felt was a neutral visual signal for insects and slowly released the chemicals for a week (unpublished). After impregnation with either synthetic blend, the felt pieces were placed inside sticky cylinders in a field experiment that tested: 1) trap odour blend, 2) floral odour blend, 3) the combination of trap and floral odour blends and 4) a hexane control with no odour. Each clear sticky cylinder (8-cm diameter × 21-cm high) was made of plastic sheet vertically positioned about 50-cm above ground on a 60-cm wooden pole. Five treatments were tested in each of five rows in a randomized block design. The sticky cylinders were spaced 3 m apart in each row while rows were separated by 5 m. Catch was recorded weekly from traps in the field (October-November 2012).

### Colour and odour in resolving pollinator-prey conflict in *D. auriculata*

Paired pink and green discs (20-mm diameter and 3.3-mm thickness made of polypropylene sheet coated with sticky polymers) were positioned 15 cm above the ground using wooden sticks (3-mm diameter) in order to mimic the visual cues of flower and trap respectively. Both discs were placed horizontally at the same height above ground and separated by a distance of 20 mm. A pair of clear plastic discs (20-mm diameter) coated with sticky polymers were separated by 20 mm at the same height and position as the coloured discs as a negative control to investigate the role of chemical cues. In this experiment, four treatments were tested: 1) pink disc and green disc without odour (to investigate the importance of visual cues only), 2) pink disc with floral odour and green disc with trap odour (to investigate the interaction of visual and chemical cues emitted from the plants), 3) clear disc with floral odour and clear disc with trap odour (to investigate the importance of chemical cues alone), and 4) a pair of clear discs without odour were used as a blank treatment (to test for passive catch levels). Each of the treatments was placed at random in a row for a total of ten rows. Disc pairs were separated by 3 m in each row, while rows were separated by 5 m (October-November 2011). The reflectance spectra of the petals and traps of *D. auriculata* as well as the pink and the green model discs were measured under natural light (Fig. 3S) using the S2000 spectrophotometer described above. The light pink discs reflected light in wavelengths similar to the *D. auriculata* flowers.

### Statistical data analysis

The mean distances between flower and nearest trap were analysed by one-way analysis of variance (ANOVA). We tested for differences in flower visitor escape frequencies from the traps of the three plant species with a chi-square test. The preferences of flower visitors and other insects for colours or for odour blends were tested by two-way ANOVA on square root transformed counts. When ANOVA was significant, pairs of treatments were compared by least squares means differences with Tukey’s honest significant difference (α = 0.05). Differences in capture frequencies between red and white discs representing spatial separations between flower and trap of *D. spatulata* and *D. arcturi* were analysed by Fisher’s exact test. Differences between captures on disc pairs of pink and green (no odours), pink (floral odour) and green (trap odour), clear (floral) and clear (trap), or clear (no odour) and clear (no odour) were analysed by exact binomial tests conditioned on equal frequencies. Statistical analyses were performed by JMP statistical software^23^.

## Additional Information

**How to cite this article**: El-Sayed, A. M. *et al.* Pollinator-prey conflicts in carnivorous plants: When flower and trap properties mean life or death. *Sci. Rep.* **6**, 21065; doi: 10.1038/srep21065 (2016).

## Supplementary Material

Supplementary Information

## Figures and Tables

**Table 1:** Floral and trap headspace volatiles identified from the sundew, *Drosera auriculata.*

Compound	RI^1^	Relative amounts (%)^2^	
		Flower	Trap
(+/−)-α -Pinene	932	2.14 ± 0.5	
Benzaldehyde	964	5.15 ± 1.4	
(−)-β-Pinene	977	6.76 ± 2.2	
(+/−)-Limonene	1030	4.34 ± 0.73	
Benzyl alcohol	1038	13.8 ± 3.1	
Phenylacetaldehyde	1046	3.22 ± 0.51	
Linalool	1101		4.61 ± 1.21
2-Phenylethanol	1116	30.27 ± 2.5	
2′-Aminoacetophenone	1307	34.28 ± 0.5	
Geranyl acetone	1450		15.08 ± 4.43
(*E*)-beta-Farnesene	1454		5.93 ± 1.65
Plumbagin	1612		74.37 ± 4.77

^1^Kovats Retention Index (VF5-MS capillary column).

^2^Percentage of total volatiles produced are given as mean areas of the GC peaks followed by the standard errors (n = 10).
