# Supplementary material for: Pollinator-prey conflicts in carnivorous plants: When flower and trap properties mean life or death
Source: Sci Rep. 2016 Feb 18;6:21065. doi: 10.1038/srep21065 (PMC4757879; doi:10.1038/srep21065)
Supplement: Supplementary Information [file srep21065-s1.doc]

**Supplementary materials**

**Pollinator-prey conflicts in carnivorous plants: When flower and trap properties mean life or death**

Ashraf M. El-Sayed^1*^, John A. Byers^2^, and David M. Suckling^1,3^

^1^*The New Zealand Institute for Plant & Food Research Limited
Gerald Street, 7608
Lincoln, New Zealand*

*^2^Department of Entomology*

*Robert H. Smith Faculty of Agriculture, Food and Environment*

*The Hebrew University of Jerusalem*

*Rehovot, Israel*

*^3^School of Biological Sciences
University of Auckland
Tamaki Campus, Building 733
Auckland, New Zealand*

^*^Author for correspondence email: [*ashraf.el-sayed@plantandfood.co.nz*](mailto:ashraf.el-sayed@plantandfood.co.nz)


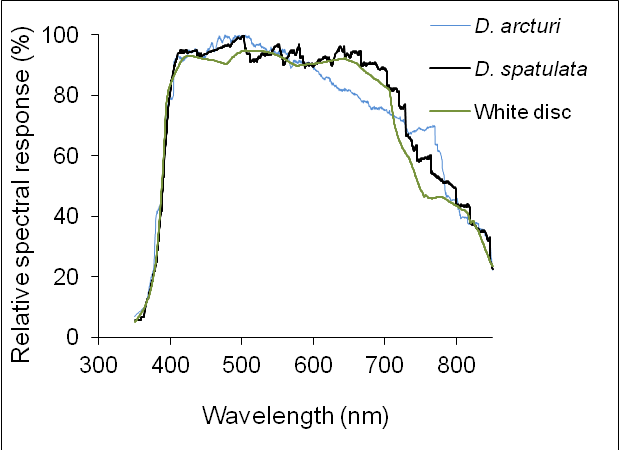


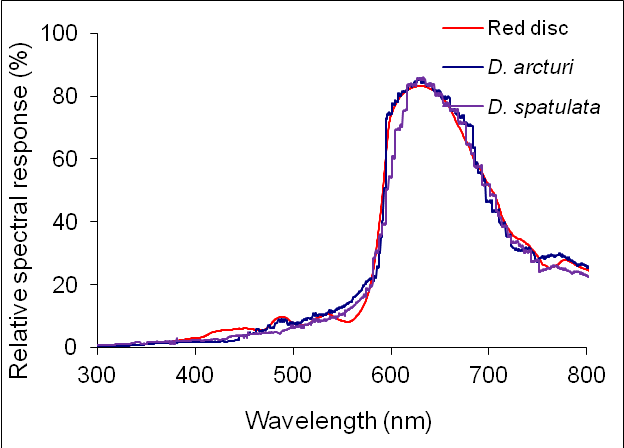


**Fig. 1S.** Spectral distribution of reflection from flowers of *D arcturi*, *D. spatulata* and white disc (top); from traps of *D arcturi*, *D. spatulata* and red disc (bottom).


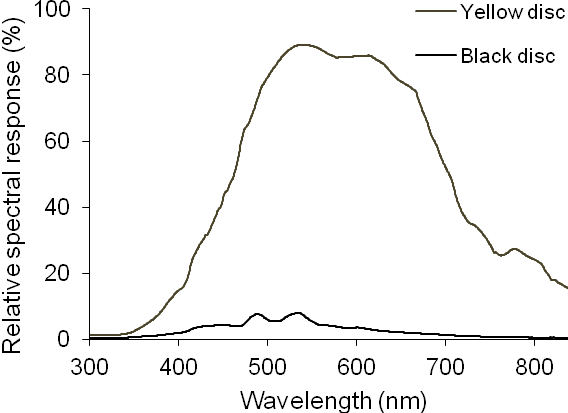


**Fig. 2S.** Spectral distribution of reflection from yellow and black discs used in field trials (spectral distribution of reflection from other colours are provided with respective species).


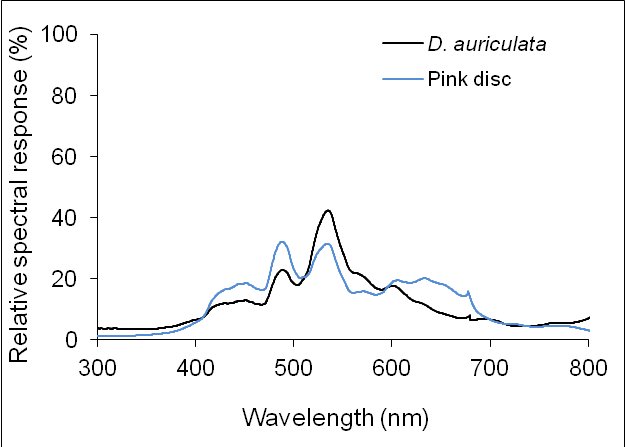


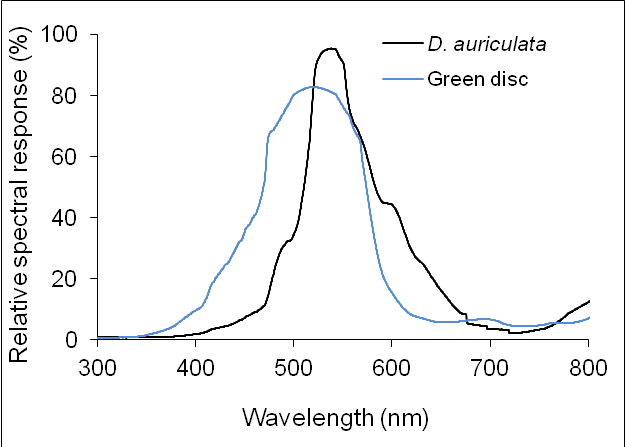


**Fig. 3S.** Spectral distribution of reflection from flowers of *D auriculata* and pink disc (top); from traps of *D auriculata* green disc (bottom).


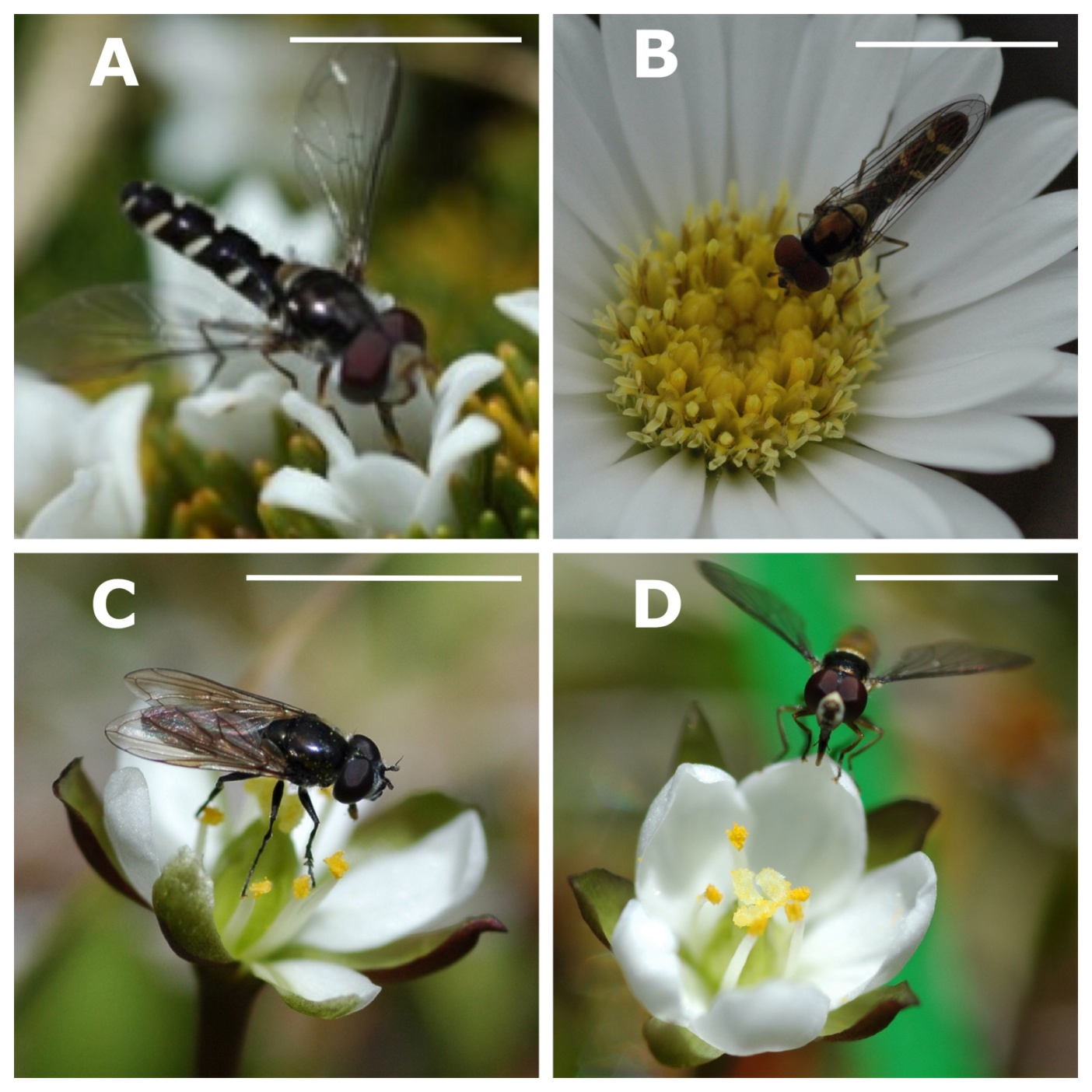


**Fig. 4S.** Syrphid flower visitors of *Donatia novae-zelandiae* (A), and *Celmisia gracilenta* (B) that share the same habitat with the two sundew species *Drosera spatulata* (C), *Drosera arcturi* (D). Scale bar = 1 cm.
